# Beyond the Watershed: Chronic Ischemic Colitis Masking Underlying Small Bowel Non-Hodgkin Lymphoma

**DOI:** 10.7759/cureus.99616

**Published:** 2025-12-19

**Authors:** Hira Khan, Yara Hamadah, Ajay Jani, Adail Dsouza, Jennifer Baldwin

**Affiliations:** 1 Internal Medicine, University of Connecticut Health, Farmington, USA; 2 Internal Medicine, University of Connecticut School of Medicine, Farmington, USA

**Keywords:** endoscopy, gastroenterology and endoscopy, gastrointestinal lymphoma, ischemic colitis, right-sided colitis

## Abstract

Ischemic colitis (IC) most commonly affects the watershed regions of the left colon, whereas right-sided involvement is less common but associated with higher morbidity, mortality, and a greater likelihood of requiring surgery. Atypical presentations, especially when driven by underlying pathology, demand heightened clinical suspicion. We present a case of right-sided colonic ischemia in a patient with brief hemodialysis exposure who was later found to have aggressive B-cell lymphoma involving the bowel.

A 64-year-old woman with hypertension, hyperlipidemia, prior cerebrovascular accident, peripheral arterial disease, chronic gastroesophageal reflux disease, and autosomal dominant polycystic kidney disease presented with one month of intermittent crampy abdominal pain. In 2024, she had undergone outpatient hemodialysis for three months before receiving a kidney transplant.

On admission, her vital signs were stable. Laboratory evaluation revealed leukocytosis (15.2 × 10³/μL), hemoglobin 12.0 mg/dL, baseline creatinine 1.8 mg/dL, and normal lactic acid. Computed tomography of the abdomen and pelvis showed mural thickening of the cecum and ascending colon with pericolonic stranding. Colonoscopy demonstrated diffuse friability and continuous ulceration in the proximal colon concerning for right-sided IC. Exploratory laparotomy revealed an ischemic, perforated cecum, an omental mass, and small bowel tumors. Right hemicolectomy with end ileostomy was performed, and pathology confirmed high-grade B-cell lymphoma. She required intensive care with vasopressors and mechanical ventilation and was treated for cytomegalovirus viremia with intravenous ganciclovir followed by valganciclovir. Gastric biopsy confirmed diffuse large B-cell lymphoma with c-Myc rearrangement.

Right-sided IC carries increased risk of transmural necrosis and sepsis, particularly in patients with vascular disease, immunosuppression, or prior dialysis. Primary intestinal lymphoma can mimic IC through obstruction and microvascular compromise. Early recognition of atypical right-sided IC is essential, as prompt surgery and biopsy can identify underlying malignancy and guide timely oncologic management.

## Introduction

Ischemic colitis (IC) is the most common form of intestinal ischemia, typically affecting older adults and those with underlying vascular disease. It most commonly involves the watershed regions of the left colon, where perfusion is most vulnerable. Right-sided involvement is much less frequent, reported in approximately 10-25% of IC cases, but is associated with significantly higher morbidity and mortality, often requiring surgical intervention [1]. This more severe clinical course is attributed to the right colon’s limited collateral circulation and its greater susceptibility to systemic hypoperfusion [2].

Early diagnosis is essential yet challenging. The diagnostic evaluation of suspected IC integrates clinical presentation, laboratory markers (such as leukocytosis or elevated lactate), and cross-sectional imaging, which may show bowel wall thickening, pericolonic stranding, or signs of impending ischemia [3]. Colonoscopy remains the gold standard for diagnosis, allowing direct visualization of mucosal edema, ulceration, or necrosis and providing tissue for histopathologic confirmation. However, in atypical cases, particularly those involving the right colon, endoscopic findings may overlap with infectious, inflammatory, or infiltrative conditions, necessitating a broad differential diagnosis [4].

Secondary or contributing conditions such as malignancy, hematologic disorders, prior hemodialysis, and immunosuppression may further compromise mesenteric perfusion and lead to atypical presentations of IC [5]. Gastrointestinal lymphomas, though rare, can mimic ischemia and may only be identified after surgical exploration or biopsy.

We present a case of right-sided colonic ischemia in a patient who was briefly on hemodialysis and was later found to have aggressive B-cell lymphoma of the bowel. Identifying atypical presentations is critical, particularly when underlying pathology contributes to disease severity.

## Case presentation

A 64-year-old woman with hypertension, hyperlipidemia, prior cerebrovascular accident, peripheral arterial disease, chronic gastroesophageal reflux disease, and autosomal dominant polycystic kidney disease presented to the hospital from home with a one-month history of intermittent abdominal pain. In 2024, she started on outpatient hemodialysis, which continued for three months before receiving a kidney transplant. Her post-transplant course was complicated by cytomegalovirus (CMV) infection and BK viremia. Her outpatient medications included prednisone, tacrolimus, aspirin, simethicone, atorvastatin, and pantoprazole.

She reported intermittent diffuse abdominal pain that was cramping in nature with no association of pain with meals. There were no exacerbating factors that the patient could identify, and the pain subsided spontaneously without analgesics within a few minutes of onset. She had no unintentional weight loss, changes in appetite, nausea, vomiting, constipation, or diarrhea during this time. She underwent screening colonoscopy in 2023, which revealed no abnormalities and was recommended a 10-year recall. She was seen in the primary care setting for abdominal pain and was prescribed simethicone due to suspicion of irritable bowel syndrome.

Vitals on admission were notable for blood pressure of 130/75 mmHg, heart rate of 86 bpm, and oral temperature of 97.5°F (36.4 °C). Physical exam on admission revealed mild generalized abdominal tenderness and no signs of peritonitis. Laboratory studies are shown in Table 1.

**Table 1:** Laboratory results with reference ranges. WBC, white blood cell count; Hgb, hemoglobin; Cr, creatinine; Lactate, lactic acid; CMV, cytomegalovirus; PCR, polymerase chain reaction.

Test	Result	Reference Range
White Blood Cell Count (WBC)	15.2 × 10³/μL	4.0–11.0 × 10³/μL
Hemoglobin (Hgb)	12.0 g/dL	12.0–16.0 g/dL (female)
Creatinine (Cr)	1.8 mg/dL	0.6–1.1 mg/dL (female)
Lactic Acid (Lactate)	0.7 mmol/L	0.5–2.2 mmol/L
Cytomegalovirus PCR (CMV PCR)	4322 IU/mL (positive)	Negative/undetectable

**Figure 1:**
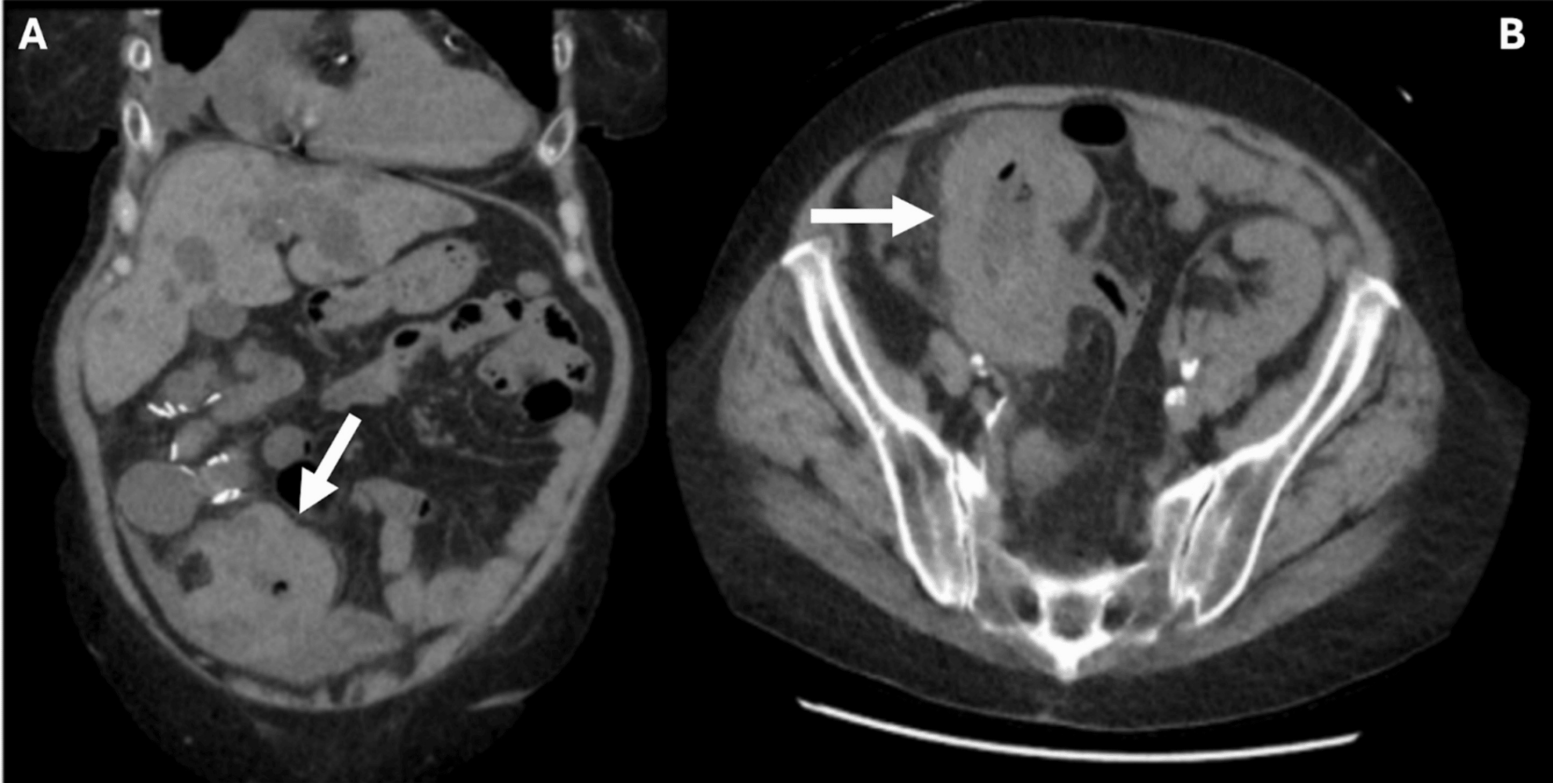
(A and B) Contrast-enhanced CT of the abdomen and pelvis (coronal and axial) demonstrating marked mural thickening of the cecum and ascending colon with pericolonic stranding.

Contrast-enhanced CT of the abdomen and pelvis was done, which demonstrated marked mural thickening of the cecum and ascending colon with pericolonic stranding as seen in Figures 1A, 1B. She was also incidentally found to have cardiomegaly, moderate pericardial effusion, and moderate pelvic ascites of unclear etiology.

Hospital course

**Figure 2:**
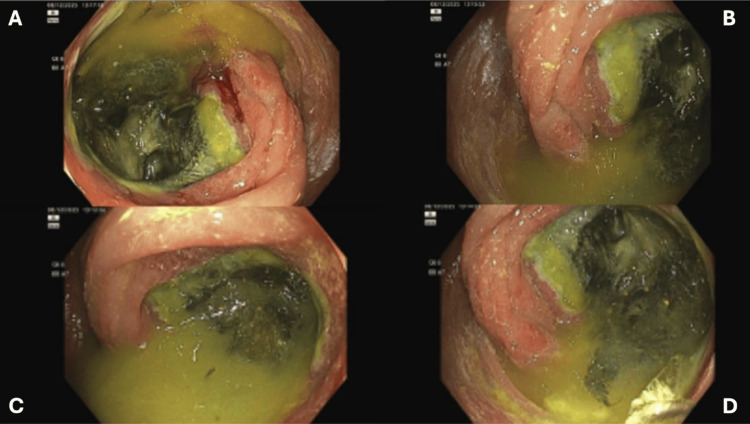
Colonoscopy images of ischemic and ulcerated mucosa in the cecum

In-patient colonoscopy was done, which showed diffuse friability throughout the colon, with a continuous segment of ulcerated mucosa bearing adherent slough and stigmata of recent bleeding in the proximal ascending colon and cecum, raising concern for right-sided IC (Figure 2). Biopsies were taken throughout the colon and from the edge of the ulcerated mucosa. Colorectal surgery was informed regarding our colonoscopy findings, and the patient was transferred to another facility where a transplant team was available for further management of the patient’s complex co-morbidities.

The patient underwent exploratory laparotomy, which showed an ischemic cecum with perforation, an omental mass, and two small bowel masses (one at the jejunum and one smaller mass in the terminal ileum). Right hemicolectomy and end ileostomy were performed; however, due to increasing vasopressor requirements, the decision was made to leave the bowel in discontinuity. The masses were sent for histopathology. She was transferred to the Intensive Care Unit (ICU), intubated and sedated, with dual pressor support and an open-abdomen negative pressure therapy system in place, with plans to return to the OR in 24 hours. Antibiotic coverage with IV meropenem, IV piperacillin-tazobactam, and IV micafungin was started, given her critical condition. Immunosuppressive therapy with IV methylprednisolone (Solu-Medrol) was continued while the patient remained NPO.

She then returned to the OR during which a small bowel anastomosis was done and an ostomy was created. During her ICU stay, she was weaned off pressor support and extubated without complication. Unfortunately, she developed high output from her ostomy, and given her immunocompromised status, there was a concern for cytomegalovirus infection. CMV PCR was positive at 4322 IU/ml, and she received IV ganciclovir, which was later transitioned to daily valganciclovir with resolution of symptoms. Antibiotic coverage was changed to oral cefazolin, metronidazole and IV meropenem was continued.

She underwent paracentesis, and fluid analysis showed no evidence of active infection. A transthoracic echocardiogram was done, which revealed a stable moderate pericardial effusion with no tamponade physiology. The etiology of her pericardial effusion remained unclear, and drainage carried a high risk due to its technically challenging posterior location.

During this time, histopathological examination of the small and large bowel lesions from colonoscopy biopsies revealed aggressive B-cell non-Hodgkin lymphoma of the bowel. Biopsies from the exploratory laparotomy corroborated this, as they showed morphologic atypia of the cells within the submucosal infiltrate, in combination with the expansile collections of CD20+ B-cells outside of follicles. The elevated proliferation index suggested that the lesion was high-grade B-cell lymphoma.

Hematology and Oncology was consulted for a newly diagnosed aggressive B-cell lymphoma, and the patient was started on allopurinol for tumor lysis syndrome prophylaxis. The patient remained in the hospital for 14 days, during which her diet was advanced, and underwent aggressive physical therapy. She was discharged to a skilled nursing facility with outpatient follow-up for chemotherapy. The outpatient PET scan revealed intense metabolic uptake involving the body of the stomach, which prompted upper endoscopy.

**Figure 3:**
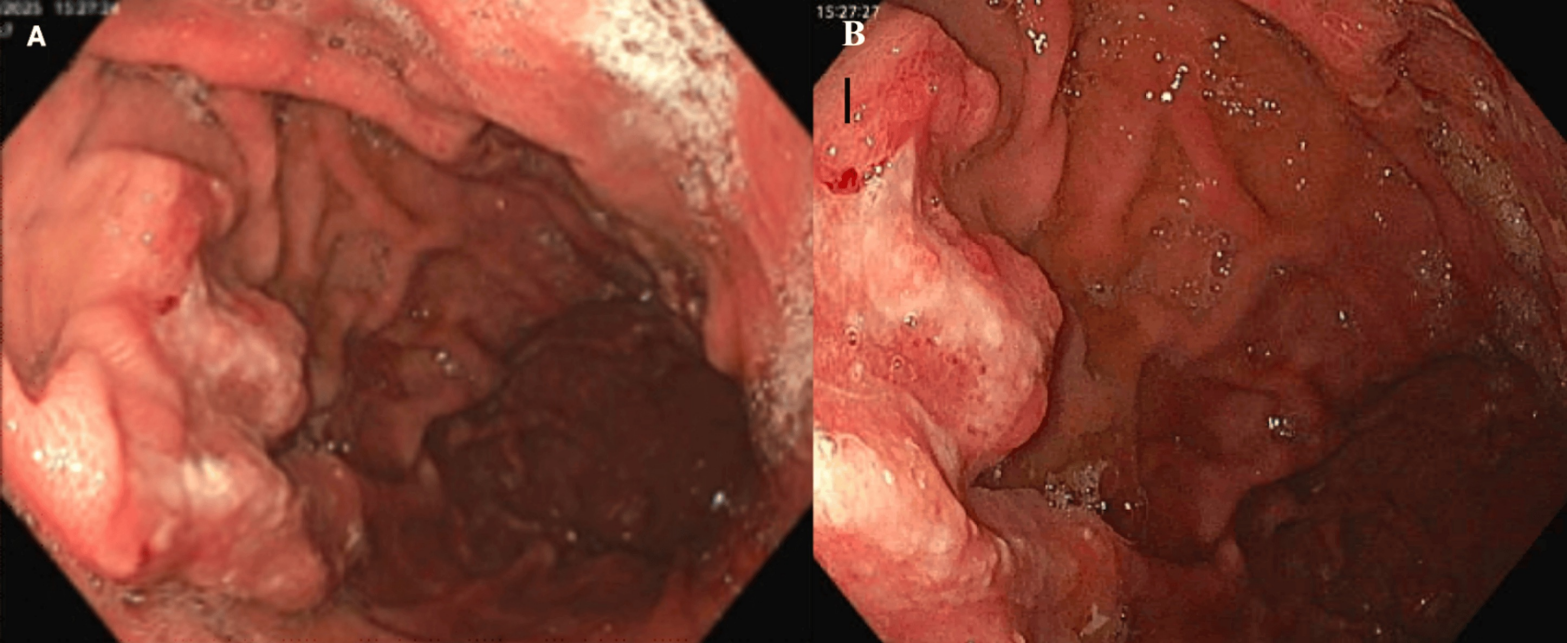
Images from esophagogastroduodenoscopy (EGD) showing an ulcerated mass in the gastric fundus

Upper endoscopy showed a large ulcerated mass in the gastric fundus, and biopsies were taken with cold forceps (Figures 3A, 3B). Histopathology confirmed atypical lymphoid infiltrate in the lamina propria, consisting of large atypical lymphocytes with multiple prominent-to-inconspicuous nucleoli. Fluorescence in situ hybridization (FISH) analysis showed cells positive for CD20, CD10, BCL2, BCL6, and MUM1. A c-Myc gene rearrangement was also detected. The findings were consistent with diffuse large B-cell lymphoma (DLBCL). An immunoperoxidase stain was negative for Helicobacter pylori.

The patient has since begun outpatient chemotherapy under the care of hematology/oncology. He continues to follow closely with the oncology team, with treatment response and surveillance plans currently underway.

## Discussion

IC typically affects elderly patients with vascular comorbidities and most often involves the left colon, particularly the splenic flexure and sigmoid colon, which are watershed areas with limited collateral circulation. In contrast, right-sided colonic ischemia is uncommon but carries a substantially higher risk of transmural necrosis, sepsis, and mortality compared to left-sided disease [1]. This pattern reflects the vascular anatomy of the right colon, which depends on the superior mesenteric artery and may be more vulnerable to systemic hypoperfusion, vasospasm, or microvascular compromise [2].

In this case, the patient’s multiple risk factors, including hypertension, peripheral arterial disease, prior hemodialysis, and recent renal transplantation, likely predisposed her to colonic ischemia. Transient episodes of hypotension during dialysis or post-transplant immunosuppressive therapy may have further impaired mucosal perfusion [5]. Additionally, chronic kidney disease and vascular calcification are known to diminish intestinal perfusion reserve, which could explain the ischemic injury localized to the right colon [3].

Primary intestinal lymphomas are uncommon, comprising 10%-20% of gastrointestinal (GI) lymphomas and account for about 0.2%-1% of all colonic malignancies [6,7]. These tumors are predominantly NHL and are usually observed in immunosuppressed patients. The stomach is the most common site of GI NHLs, accounting for 68-75% of the cases, followed by the small intestine (15-20%), with the remainder being in the colon, esophagus, and rectum [6]. Among histologic subtypes, DLBCL is the most common and often involves the right side of the colon, likely due to the abundance of lymphoid tissue [8].

Common presenting symptoms of intestinal lymphomas include abdominal pain, weight loss, change in bowel habits, and anemia [7]. Neoplastic infiltration of the bowel wall can lead to luminal narrowing, microvascular obstruction, and mucosal necrosis, mimicking or precipitating IC [9]. Moreover, the associated lymphomatous mass effect or mesenteric vascular involvement may further compromise regional blood flow, leading to perforation and severe ischemic injury, as seen in our patient [9].

Right-sided IC secondary to underlying malignancy is rare but clinically significant because it often presents without classic features such as hematochezia, and with inadequate immune response, it can lead to delayed recognition with patient mortality [10]. In this case, the patient’s symptoms were nonspecific, and the initial suspicion of irritable bowel syndrome delayed definitive imaging and intervention. Recognition of atypical or persistent right-sided abdominal pain in high-risk patients, especially those with immunosuppression, should prompt early cross-sectional imaging, usually with a contrast-enhanced CT, which would provide information about tumor size, invasion depth, and local lymph node metastasis, if intestinal lymphoma is present [11].

The diagnosis of intestinal lymphoma relies on histopathologic and immunohistochemical analysis following endoscopic or surgical biopsy. Histopathology remains the gold standard for diagnosis in such cases, and CD20 positivity with a high Ki-67 proliferation index confirmed a high-grade B-cell lymphoma [7]. Timely identification of this underlying pathology is essential, as management strategies differ markedly from those of primary ischemic injury.

The current standard of care for DLBCL (including gastric DLBCL) is immunochemotherapy with rituximab combined with CHOP (cyclophosphamide, doxorubicin, vincristine, and prednisone). The 2022 POLARIX trial compared the addition of Polatuzumab Vedotin to R-CHP (Pola-R-CHP) and demonstrated superior progression-free survival compared to R-CHOP, establishing it as a potential alternative first-line regimen, particularly in intermediate and high-risk disease [12]. In gastric DLBCL with Helicobacter pylori positive disease, initial H pylori eradication may induce remission in select cases, emphasizing the importance of testing for the microorganism at the time of diagnosis [13].

The discovery of aggressive B-cell lymphoma within the bowel represents a critical diagnostic turning point. Primary GI lymphomas account for only 1-4% of all GI malignancies, and the ileocecal region is the most frequently involved site [6]. Neoplastic infiltration of the bowel wall can lead to luminal narrowing, microvascular obstruction, and mucosal necrosis, mimicking or precipitating IC [9]. Moreover, the associated lymphomatous mass effect or mesenteric vascular involvement may further compromise regional blood flow, leading to perforation and severe ischemic injury, as seen in our patient.

This case highlights several important learning points. Right-sided IC should prompt consideration of underlying systemic or infiltrative processes, especially in patients with atypical presentations or immunosuppression. Hemodynamic instability and prior renal replacement therapy further increase vulnerability by compromising mesenteric perfusion. Additionally, bowel lymphoma can mimic IC, given overlapping radiologic and endoscopic features; therefore, clinicians should maintain a high index of suspicion for malignancy when ischemic lesions demonstrate unusual distribution, marked mucosal friability, or mass effect on imaging.

## Conclusions

This case underscores the importance of recognizing atypical presentations of IC, particularly chronic right-sided disease, which carries a higher risk of complications and mortality. Furthermore, it highlights the need to consider underlying malignancy as a potential contributing factor, especially in patients with complex medical histories. Furthermore, underlying malignancy and immunocompromised state should be considered as a potential contributor. Timely diagnosis and surgical intervention can be life-saving and may reveal critical pathology that significantly influences prognosis and management. 
